# An Unusual Cause of Coma

**DOI:** 10.1155/2012/982198

**Published:** 2012-09-29

**Authors:** Sujith Ovallath, R. S. Raj, Abdu Rahman, A. S. Girija

**Affiliations:** ^1^Kannur Medical College, Kerala, Kannur 670612, India; ^2^Government Medical College, Calicut 673008, India

## Abstract

This case report is an unusual presentation of fibroid uterus as coma. The patient developed a recurrent hypoglycemia possibly secondary to the insulin-like growth factor secreted from the fibroid. The hypoglycemic symptoms disappeared on removal of the fibroid. The histopathological examination revealed no evidence of malignancy.

## 1. An Unusual Cause of Coma

A 37-year-old female presented in coma of gradual onset. Her family reported episodic abnormal behavior in the mornings, excessive sleepiness, and episodic vomiting of one-week duration prior to the onset of coma. She was initially seen by a psychiatrist and referred for evaluation. While being in the hospital, she had repeated episodes of seizures. There was no history of toxic drug or alcohol ingestion. Initial evaluation: patient was in coma (E2 M3 V2). Pupils were middilated. She was afebrile, excessively sweating, having no neck stiffness, bilateral upper limb rigidity, and her fundus was normal and plantars were extensor. 

Her investigations showed random blood sugar—54 mg%, blood urea—43 mg%, serum creatinine—0.9 mg%, serum electrolytes—Na 131 meq/L, K 3.5 meq/L, peripheral smear—negative, no malarial parasites. Liver function test—normal, CSF microscopy, protein sugar, culture, and CSF PCR for herpes were normal, serum calcium and phosphorus—normal, CPK—normal, ECG—normal, EEG—normal, plasma insulin level—normal, CT Scan—showed diffuse brain oedema. 

MRI brain showed patchy hyper intensities in pones medulla superior cerebellar peduncle and hippocampus in flair and T2 W images (see Figure 2). MR venogram was normal.

An ultrasound abdomen showed a large fibroid otherwise normal.

## 2. Treatment and Hospital Course

 Initial clinical diagnosis was viral encephalitis. But her blood sugar values were repeatedly showing low values especially fasting early morning values. On dextrose administration, patient made gradual improvement over days. After improvement she had repeated early morning hypoglycemic symptoms and documented low blood sugar values. An extended fasting and C-peptide estimation was done which showed serum C-peptide <0.50 (0.9–4) Ng/mL.

**Figure 1:**
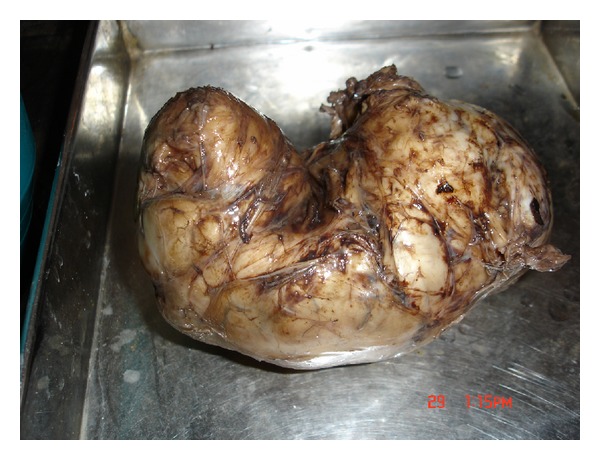
The fibroid.

**Figure 2:**
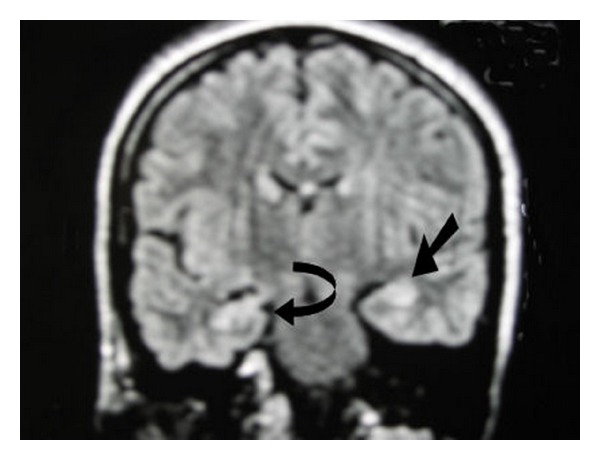
MRI showing findings suggestive of hypoglycemia.

 The possibilities considered were insulinoma or exogenous insulin-like substance [1]. She was suggested octreotide scintigraphy and CT abdomen. CT abdomen did not revealed any pancreatic mass, but she could not afford octreotide scintigraphy. So a clinical possibility fibroid uterus secreting insulin-like substance was thought of and the removal of large fibroid was suggested to see the response. The removed tumor showed no evidence of malignancy. She had no further attacks of hypoglycemia after the removal of the tumor (see Figure 1). 

## 3. Discussion 

The most possible cause of recurrent hypoglycemia in this case is insulin-like growth factor secretion from fibroid which is confirmed by the improvement of hypoglycemia after removal of the tumor. Nonislet cell tumor hypoglycemia (NICTH) due to the production of an aberrant form of insulin-like growth factor 2 (IGF 2) [2] is a well-recognised phenomenon [3, 4]. IGF I level falls due to negative feedback. Hypoglycemia results from IGF II action through insulin or IGF I receptors. In insulinoma patients, C-peptide levels are not suppressed [5].
